# Vector Competence of *Culex quinquefasciatus* from Brazil for *West Nile Virus*

**DOI:** 10.3390/tropicalmed8040217

**Published:** 2023-04-06

**Authors:** Lúcia Aline Moura Reis, Eliana Vieira Pinto da Silva, Daniel Damous Dias, Maria Nazaré Oliveira Freitas, Rossela Damasceno Caldeira, Pedro Arthur da Silva Araújo, Fábio Silva da Silva, José Wilson Rosa Junior, Roberto Carlos Feitosa Brandão, Bruna Laís Sena do Nascimento, Lívia Caricio Martins, Joaquim Pinto Nunes Neto

**Affiliations:** 1 Graduate Program in Parasitary Biology in the Amazon Region, Center of Biological and Health Sciences, State University of Pará, Belém 66095-663, Brazil; 2 Department of Arbovirology and Hemorrhagic Fevers, Evandro Chagas Institute—IEC/MS/SVSA, Ananindeua 67030-000, Brazil; 3 Graduate Program in Biology of Infectious and Parasitary Agents, Biological Sciences Institute, Federal University of Pará, Belém 66077-830, Brazil

**Keywords:** *West Nile virus*, flavivirus, *Culex quinquefasciatus*, arbovirus infections

## Abstract

*West Nile virus* is characterized as a neurotropic pathogen, which can cause West Nile fever and is transmitted by mosquitoes of the genus *Culex*. In 2018, the Instituto Evandro Chagas performed the first isolation of a WNV strain in Brazil from a horse brain sample. The present study aimed to evaluate the susceptibility of orally infected *Cx. quinquefasciatus* from the Amazon region of Brazil to become infected and transmit the WNV strain isolated in 2018. Oral infection was performed with blood meal artificially infected with WNV, followed by analysis of infection, dissemination, and transmission rates, as well as viral titers of body, head, and saliva samples. At the 21st dpi, the infection rate was 100%, the dissemination rate was 80%, and the transmission rate was 77%. These results indicate that *Cx. quinquefasciatus* is susceptible to oral infection by the Brazilian strain of WNV and may act as a possible vector of the virus since it was detected in saliva from the 21st dpi.

## 1. Introduction

Arboviruses are viruses that complete part on their life cycle in arthropod vectors and thus are transmitted to vertebrates [1,2]. The life cycle of arboviruses involves the feeding on a viremic animal by a hematophagous arthropod, followed by the replication of the virus in the arthropod. The virus can be transmitted to other animals and humans when it reaches the salivary glands [1,3,4].

WNV was first isolated in 1937 from a human sample in West Nile District, Uganda (strain B956, lineage 2) [5]. In Brazil, the Evandro Chagas Institute (IEC) performed the first viral isolation from a horse brain sample from Pedra Grande region on the São Mateus municipality, Espírito Santo state, Brazil, in 2018 [6], and phylogenetic analysis demonstrated that this WNV strain belongs to the 1A lineage that circulates in the United States and Mexico [6,7]. As of July 2019, only two human cases have been confirmed, both in the state of Piauí, between 2014 and 2017, according to the Ministry of Health’s (MOH) West Nile surveillance report [8].

The WNV is characterized as a neurotropic pathogen that causes the West Nile fever, febrile illness, encephalitis, and also can cause asymptomatic infections [9]. It is transmitted by mosquitoes, mainly of the *Culex* genus [10], and can also be transmitted through contact with blood and tissues from infected animals [11] and through organ transplants [12,13], blood transfusions [14,15], and the transplacental pathway [16].

The enzootic cycle of WNV consists of hematophagous arthropods as vectors, wild birds as amplification hosts, and mammals (e.g., horses and humans) as accidental hosts [17]. Members of the *Culex* genus are accepted as the main vectors [18,19,20,21].

The *Culex quinquefasciatus* (SAY, 1823) species is mainly found in countries with warmer climates and is widely adapted to the urban environment, being easily found in human and animal dwellings [22,23]. The females oviposit the rafts of eggs in small collections of stagnant water with a high content of organic matter, making this species resistant to the effects of water pollution [22,24].

In Brazil, *Cx*. *quinquefasciatus* is a cosmopolitan mosquito and has a wide distribution. Entomological studies in the states of Pará, Mato Grosso, São Paulo, and Rio de Janeiro between 1968–1976, in Amazonas between 2002–2005 [25,26,27], and in Rio Grande do Sul between 2006 and 2008 [22,28], reported the presence of the species, including areas belonging to the Amazon Region. In a study conducted in the Brazilian Amazon [29,30,31], the largest number of identified species belonged to the genus *Culex* Linnaeus, with *Culex* (Melanoconion) *gnomatos* being the most abundant species. According to the *Cx. quinquefasciatus* Surveillance Guide [32], this species is associated with high lymphatic filariasis rates in Recife, Maceió, and Belém. WNV has been detected in 27 species of mosquitoes in the United States, including *Aedes*, *Anopheles*, *Mansonia*, and *Psorophora* mosquitoes, and 14 species of *Culex*, including *Culex quinquefasciatus*, according to the Centers for Disease Control and Prevention (CDC) [33].

*Culex* species, including *Culex tarsalis*, *Culex pipiens*, and *Culex quinquefasciatus*, are currently recognized as the primary vectors of WNV [10,33,34,35], and the vector competence of Culex *quinquefasciatus* for WNV transmission has been demonstrated in several studies [21,35,36,37]. The practice of hematophagy is common among several insects that parasitize vertebrate animals, since females use blood as a source of amino acids needed for the maturation of their eggs [38]. Vertebrate blood is rich in several nutrients, and blood feeding is not only a nutrient source for arthropods but also a rich source of infection, exposing them to a variety of pathogens including bacteria, fungi, and viruses [39,40]. Therefore, vector competence is defined as the ability of a vector to become infected with a pathogen (susceptibility), maintain it in tissues (extrinsic incubation period), and transmit it by saliva [10,34].

Therefore, the present study aims to evaluate the vector competence of the *Cx. Quinquefasciatus* mosquitoes, from the Amazon region of Brazil, to be infected and transmit the WNV strain (BEAN854747) isolated in Brazil in 2018 (GenBank: MH643887).

## 2. Materials and Methods

### 2.1. Mosquito Infection

**Figure 1:**
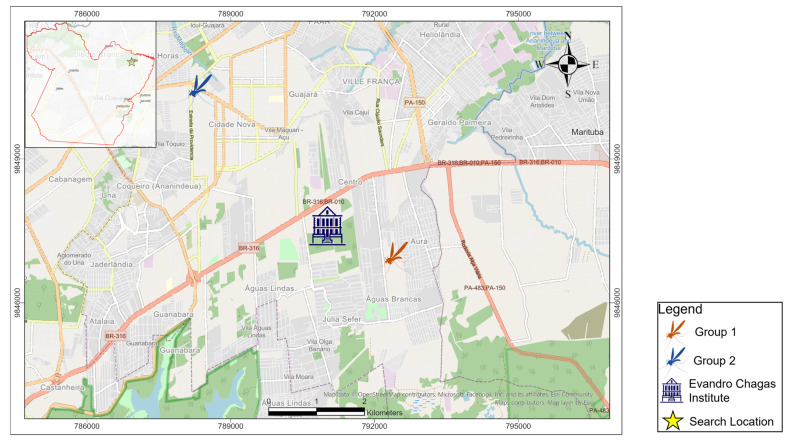
Map of the areas of origin of the generations of mosquitoes used in the study. Group 1 from the neighborhood of Águas Lindas and Group 2 from the neighborhood of Ananindeua.

Two independent experiments were conducted with colonies of *Culex quinquefasciatus* from two neighborhoods in the municipality of Ananindeua, Pará state (Northern Region). The first artificial infection experiment (Group 1) was carried out with F3 generation females from the Julia Seffer housing complex, Águas Lindas neighborhood, and the second artificial infection experiment (Group 2) was carried out with F1 generation females from the Cidade Nova neighborhood (Figure 1).

The rafts of eggs and larvae stages were reared in plastic trays containing 700 mL of distilled water, supplemented with crushed and sterilized fish feed. Pupae were transferred to a transparent polypropylene container containing 50 mL of distilled water and placed in a 30 cm^3^ insect rearing cage. Adult mosquitoes were maintained in insectary, at 28 °C ± 1 °C, humidity of 80% ± 10%, with 12:12 h light:dark cycles [35], and constantly received cotton soaked in sugar solution (10%) ad libitum (12 g of caster sugar diluted in 250 mL of water) [41].

### 2.2. Viral Strain

The BEAN854747 WNV strain (GenBank: MH643887) was isolated from the central nervous system (CNS) sample of an adult horse from Pedra Grande locality, São Mateus municipality, Espírito Santo state, Brazil. Virus isolation was performed in C6/36 cells, confirmed by indirect immunofluorescence (IF), which showed, approximately, 75% of positive cells for antibodies against flavivirus. Supernatant from C6/36 infected cells was positive for WNV by RT-PCR assay, based on protocols established by Lanciotti et al. [42] and Lanciotti and Kerst [43], and the phylogenetic analysis characterized as belonging to the 1A lineage [6].

### 2.3. Viral Stock Preparation

The WNV virus stock was prepared in Vero cells (ATCC CCL-81), in which 150 µL of virus, fifth passage, was inoculated, and incubated at 37 °C and 5% CO2 for one hour for adsorption.

After the adsorption period, 25 mL of Medium 199 (Gibco, Grand Island, NY, USA) was added; such medium contains 2% fetal bovine serum (FBS), penicillin (100 IU/mL), and streptomycin (100 µg/mL). The infected cells were incubated again for 6 days.

After identification of the cytopathic effect in 90% of the Vero cell monolayer, cell lysis was performed, by centrifugation, loosening the monolayer from the flask wall, and 10% (V/V: 2.5 mL) FBS (GIBCO) was added. Aliquots of 2 mL were placed in KMA cryogenic freezing tubes (Mylabor, Sao Paulo, Brazil) and stored at −70 °C. The aliquots to be used were thawed at ambient temperature and mixed with the blood [44,45].

The WNV viral stock was titrated by the viral titration plaque assay, obtaining a titer of 1.4 × 10^8^ PFU/mL (plaque forming units per milliliter).

### 2.4. Mosquitoes Infection

In experimental group one, 150 females were used and, in group two, 189 females; all were used from 5 to 8 days after emergence.

The females were separated and starved through sugar-deprivation for 24 h before the infected blood feeding. The oral infection was performed using a sterile glass artificial feeder connected to a water bath at 37 °C and covered with a bovine liver peritoneum membrane purchased from a slaughterhouse.

In the first experiment, the infectious blood meal was prepared by mixing 2.5 mL of defibrinated sheep blood (EBE-FARMA, Cachoeiras de Macacu, Brazil), and 1.5 mL of WNV stock. In the second experiment, 2 mL of defibrinated sheep blood and 2 mL of WNV stock were mixed. The females remained exposed to the infected blood meal for 60 min (final titer of infected blood meal: 7 × 10^7^ PFU/mL).

At the end of the oral feeding period, the females were transferred to an insect rearing cage, and a transparent polypropylene container containing 15 mL of distilled water was deposited inside the cages for oviposition [46].

In group one, we obtained 85 engorged and 65 non-engorged females that were frozen at −20 °C for 48 h and then discarded. In the second group, we obtained 108 engorged females and 81 non-engorged females that were frozen at −20 °C for 48 h and then discarded. We followed the survival of engorged females during post infection days, discarding the dead. There were 3 deaths at 6th dpi and 2 deaths at 10th dpi in the first group. In the second group, 3 females died between the 3rd and the 6th dpi, and 35 females died between the 12th and the 21st dpi. Thus, 27 females were analyzed at 7th dpi, 33 at 14th dpi, and 20 at 21st dpi. In the second group, 25 females died at 7th dpi, 23 at 14th dpi, and 22 at 21st dpi.

The control group was composed of uninfected females belonging to the same generation used in the infection fed only with uninfected blood [34,47].

### 2.5. Mosquito Segmentation

The body (thorax and abdomen) and head segmentation and saliva collection were performed on the 7th, 14th, and 21st days post-infection (dpi). Females in group 1 were segmented into head and body only, and no saliva was collected, forming pools with more than one female; in group 2, females were segmented into body, head, and saliva and samples analyzed individually.

For saliva collection, the proboscis was inserted into a 10 µL micropipette containing 5 µL of FBS (GIBCO), and after 30 min, the medium containing the saliva was transferred to Eppendorf tubes containing 45 µL of Leibovitz’s L-15 medium (GIBCO) and immediately stored at −70 °C [48,49].

For segmentation of body and head [49], the females were anaesthetized on ice, placed with the abdomen upwards on a microscope slide, and the wings and legs were removed. The body and head were separated and transferred to Eppendorf tubes, and 1000 µL of Dulbecco’s phosphate buffered saline (DPBS) (Life Technologies, Carlsbad, CA, USA) containing 2% penicillin and streptomycin, 1% fungizone, and 5% FBS was added, as well as a 3 mm stainless steel bead to perform the maceration in TissueLyser II (Qiagen, Hilden, Germany), and were stored at −70 °C [50].

### 2.6. Virus Isolation

For virus isolation, the samples were centrifuged (Mikro 220R, Hettich, Föhrenstr, Tuttlingen, Germany), and 100 µL of the macerated supernatant of the body and head samples and 20 µL from the saliva samples were inoculated in C6/36 cells (ATCC: CRL-1660) [51]. The C6/36 cells were incubated at 28 °C for one hour, and 1.5 mL of Leibovitz’s L-15 maintenance medium (GIBCO, Grand Island, NY, USA) prepared with 2.95% tryptose phosphate, nonessential amino acids, penicillin, streptomycin, and 2% SBF was added to the monolayer [45].

Inoculated cells were incubated (Napco 6100 Water Jacketed Co2 Incubator, Winchester, VA, USA) at 5% CO_2_ at 28 °C (±2 °C) and evaluated for 7 days using an inverted optical microscope (Olympus CK2 Phase Contrast Microscope, Shibuya-ku, Tokyo, Japan) to verify the occurrence of cytopathic effect.

### 2.7. Indirect Immunofluorescence Test (IF)

In the indirect immunofluorescence test (IF), 25 µL of the inoculated C6/36 cells were added to the individual holes of the immunofluorescence assay slide, then were immersed in acetone (−20 °C) for 10 min. After, 25 µL of polyclonal antibody (ratio 1:20), with hyperimmune West Nile ascitic fluid (in house) produced in adult Swiss albino mice (*Mus musculus*) by the Arbovirology and Hemorrhagic Fevers Section (SAARB/IEC), was added.

The slides were stored in a humidity chamber and in an incubator (Napco 6100 Water Jacketed Co2 Incubator) for 30 min at 37 °C and 5% of CO_2_. Next, the slides were immersed in phosphate buffered saline (PBS) pH 7.4 for 10 min, followed by washing with distilled water. After, 50 µL of fluorescein isothiocyanate-conjugated anti-mouse antibody (Cappel, catalog: 55499, FITC-conjugated goat IgG, fraction for mouse immunoglobulin IgG, IgA, and IgM, MP Biomedicals, LLC., Solon, OH, USA), diluted to a ratio of 1:900, was added to each hole, and Evans Blue (0.5%) was used as a stain [52].

The slides were again placed In a humidity chamber and in the incubator for 30 min, repeating the immersion in PBS for 10 min and finishing the slide preparation with buffered glycerin (pH 8.2) in each hole and fixing the coverslip for observation under a fluorescence microscope (Olympus BX51, uPlanFL N 20X/0.5 lens and WB and U-25nd filters).

Cells inoculated with head, body, and saliva samples from females not exposed to infective blood were used as negative controls, and the samples that had an indeterminate IF result were inoculated onto new C6/36 cells in order to increase the viral load or confirm a negative result.

Images of the samples were acquired at 200× magnification on a fluorescence microscope with a Canon PowerShot G6 camera (Canon, Tokyo, Japan).

### 2.8. Viral Titration

Positive samples were subjected to the viral titration test. In the viral titration test, 10-fold serial dilution (10^−1^ to 10^−6^) of the samples was performed in 225 µL of the Medium 199 (GIBCO) in a 96-well cell culture plate, and 25 µL of the original samples (body, head, and saliva) were added to the well, then 125 µL were aspirated and transferred to the next well, repeating this procedure until the last dilution of “−6” [53].

After the dilution process, in a 24-well plate with Vero cells (ATCC CCL-81), 100 µL of the diluted viral samples were added to each well. Subsequently, the plate was incubated for one hour, and 3 mL of carboxymethyl cellulose (CMC, 3% in medium 199) supplemented with 5% FBS, penicillin (100 UI/mL), and streptomycin (100 µg/mL) were added to each well, followed by a new incubation at 37 °C for 5 days. The cells were fixed with 3 mL of 10% formaldehyde and fixed with 3 mL of 0.1% crystal violet dye.

The viral titer was calculated by multiplying the number of plaques obtained from a given serial dilution by the dilution factor, with the result being expressed in plaque-forming units per milliliter (PFU/mL) [53].

### 2.9. Infection, Dissemination and Transmission Rates

The infection rate was calculated from the number of females with infected body among the total number of engorged females; the dissemination rate was calculated based on the number of females with an infected head among the females with an infected body; and the transmission rate was calculated according to the number of females with infected saliva among females with infected body and head [54].

### 2.10. Statistical Analysis

The analysis of infection, dissemination, and transmission rates, and the result of the IF were expressed as percentages and analyzed by the Chi-square trend test (*X*^2^) (α = 0.05) with the aim of evaluating the trend of increasing or decreasing rates. Relationships between titers in different tissues and post infection days were analyzed using Shapiro–Wilk (W) test for data distribution analysis and Kruskal–Wallis (H) and Dunn tests because the data were not normally distributed. The significance level was α = 0.05 for all tests. Statistical tests were applied using the statistical program BioEstat 5.3 (Mamirauá Institute, Belém, Brazil).

## 3. Results

### 3.1. Infection, Dissemination and Transmission Rates

**Figure 2:**
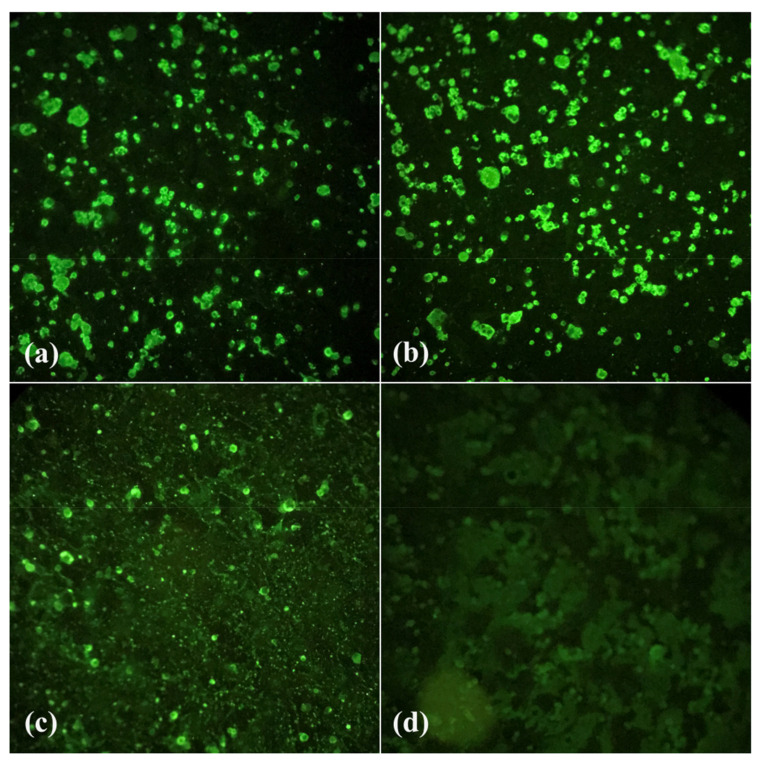
Indirect immunofluorescence of *Culex quinquefasciatus* body and head samples from Águas Lindas neighborhood (group 1). (**a**) Positive 14th dpi body sample; (**b**) Positive 21st dpi head sample; (**c**) Positive control; (**d**) Negative control. Images were taken at 200× magnification.

**Figure 3:**
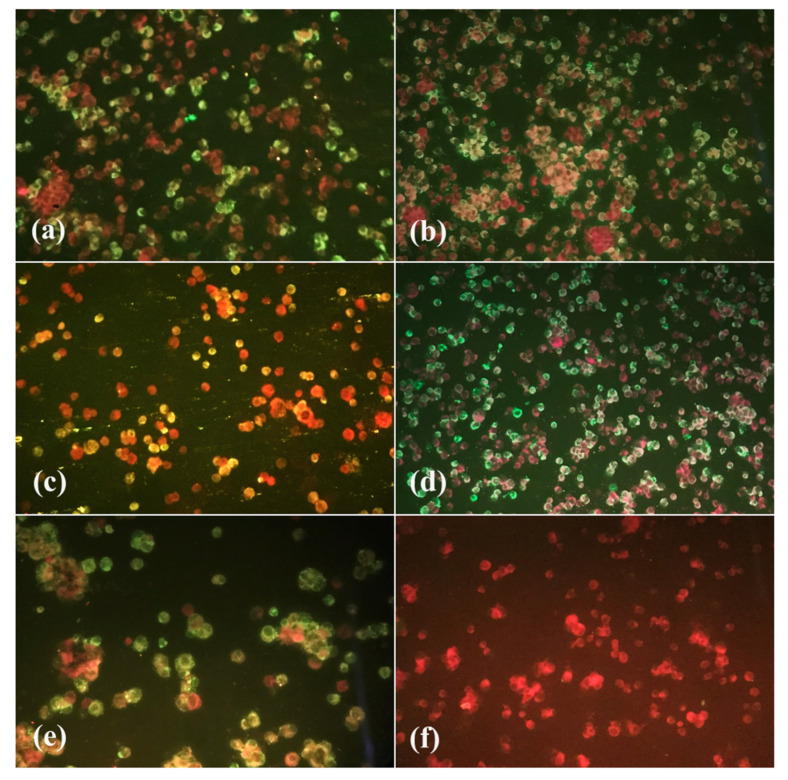
Indirect immunofluorescence of *Culex quinquefasciatus* body, head, and saliva samples from Cidade Nova neighborhood (group 2). (**a**) Positive 7th dpi body sample; (**b**) positive 21st dpi body sample; (**c**) positive 21st dpi head sample; (**d**) positive 21st dpi saliva sample; (**e**) positive control; (**f**) negative control. Images were taken at 100× magnification.

From the two artificial infection experiments, 150 females were analyzed, 52 on the 7th dpi, 56 on the 14th dpi, and 42 on the 21st dpi (Figure 2 and Figure 3).

The infection rate in G1 was 100% in the three dpi analyzed and in G2 was 84% at 7th, 96% at 14th, and 100% at 21st dpi. Thus, the total infection rate was 92% positive bodies on the 7th dpi, 98% on the 14th dpi, and on the 21st dpi all bodies (100%) were positive for WNV infection. The Chi-square (*X*^2^) test was not performed to analyze the infection rate of G1. All dpis had 100% positivity. In G2 (*p* = 0.0344, *A* = 3.7101), there was an increasing trend in the number of positive body samples as the day post-infection increased.

**Figure 4:**
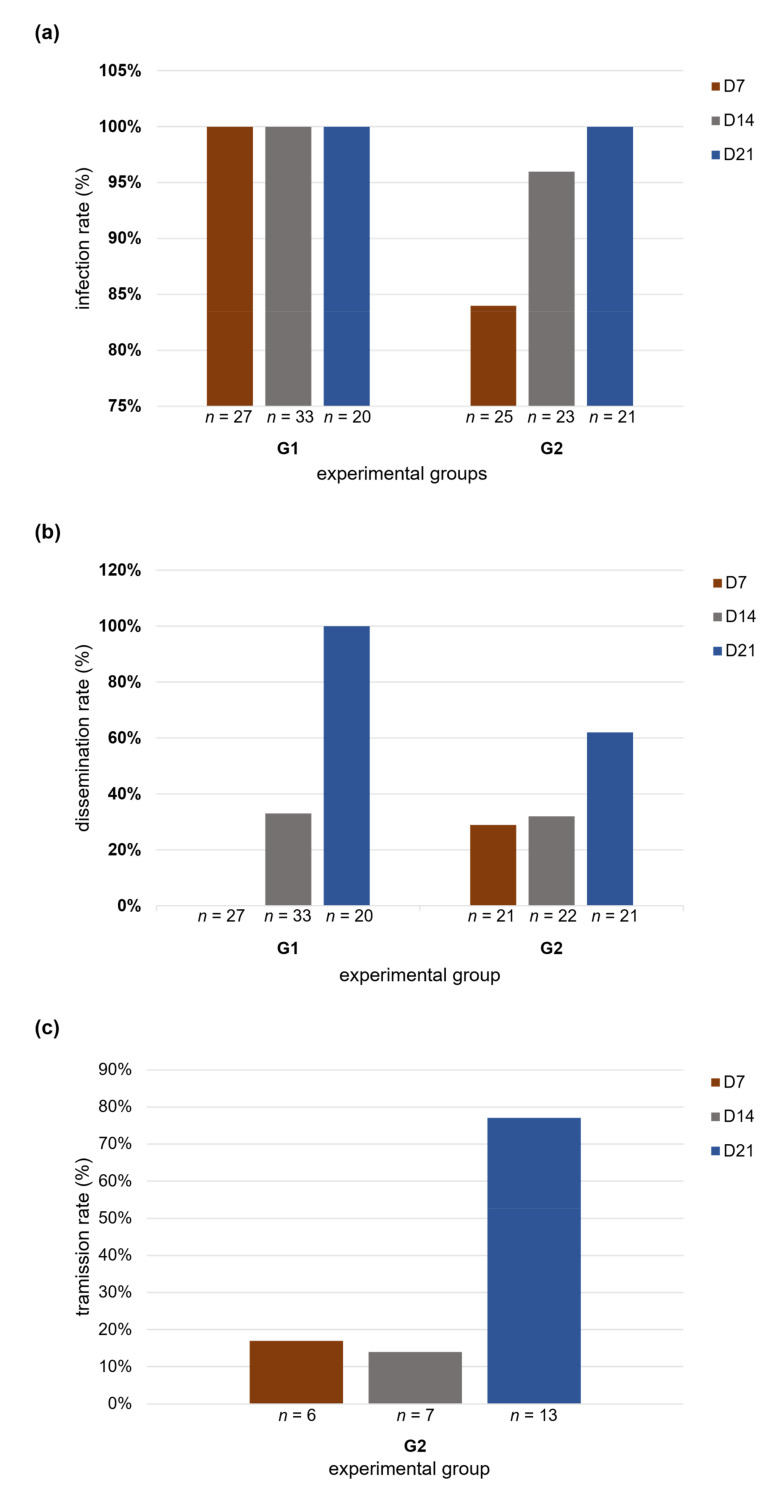
Percent parameters of vector competence of *Culex quinquefasciatus* strains from the Julia Seffer and Cidade Nova neighborhoods infected with WNV. At 7th, 14th, and 21st days after a blood meal containing WNV (7 × 10^7^ PFU/mL). Experimental groups were represented with the letter G (G1 and G2), (“*n*” indicates total samples analyzed per dpi). (**a**) Graphical representation in percentage of infection rate; (**b**) graphical representation in percentage of dissemination rate; (**c**) graphical representation in percentage of transmission rate.

The dissemination rate for G1 was 0% at 7th dpi, 33% at 14th dpi, and 100% at 21st dpi. In G2, it was 29% at 7th dpi, 33% at 14th dpi, and 62% at 21st dpi. Thus, the total dissemination rate was 13% at 7th dpi, 33% at 14th dpi, and 80% at 21st dpi (Figure 4b). *X*^2^ showed an increasing trend (G1: *p* = 0.0001, *A* = 22.7125; G2: *p* = 0.0279, *A* = 7.0000) in the number of positive head samples with increasing dpi in both groups.

Saliva infection evaluation was only for Group 2 samples. The *X*^2^ showed no trend (*p* = 0.7309, *A* = 0.6923) in the number of positive saliva samples with increasing dpi, obtaining 17% positive saliva on the 7th dpi, 14% on the 14th dpi, and 77% on the 21st dpi (Figure 4c).

### 3.2. Viral Titration

Comparative analysis of viral titers of G1 body samples showed statistical significance (*p* = 0.069, *H* = 9.9622), indicating that body viral titers were directly related to post infection day, and Dunn’s test showed a greater difference in titers between 14 and 21 dpi (*p* < 0.05). Comparative analysis of G2 body samples was not statistically significant (*p* = 0.0690, *H* = 5.3487), meaning that the variation of viral titers obtained in body samples of this group is independent of post infection day.

When comparing the variation of viral titers of head samples between dpi’s, both G1 and G2 showed no statistical significance (G1: *p* = 0.2219, *H* = 3.0111; G2: *p* = 0.0535, *H* = 5.4553), demonstrating that the variation of viral titers of such samples is independent of the post infection day analyzed.

Regarding the viral titers of the G2 saliva samples, there are no positive salivas at dpi 7. There is only one positive saliva at dpi 14 and a higher quantitative saliva at dpi 21, making a comparative analysis between post-infection days impossible. The viral titer of the 14th dpi saliva was 200 PFU/mL. On the 21st dpi, the titer ranged from 100 PFU/mL to 3 × 10^6^ PFU/mL.

## 4. Discussion

The analysis of the susceptibility of vectors to arbovirus infection is extremely important for the study of the vectorial competence of arthropods, determining their participation in the transmission cycle. Thus, we used mosquitoes from two sites in the Amazon region to identify whether they have the ability to acquire and transmit WNV.

In our work, the viral stock was produced by virus inoculation of Vero cells, which resulted in a final titer of 10^8^ PFU/mL, a relatively high titer. However, this value is similar to that used in another paper, which obtained a titer of 7.52 log_10_ of the stock also produced in Vero cells [55].

The population of *Cx. quinquefasciatus* used in the study has high susceptibility to infection by the BEAN854747 (GenBank: MH643887) strain of WNV, since our results demonstrated the presence of the virus in 92% of body samples at 7th dpi, 98% at 14th dpi, and 100% at 21st dpi, corroborating the data presented by Sudeep et al. [56], who evaluated populations of *Cx. quinquefasciatus* from India for transmission of three different strains of WNV, which also showed susceptibility of the species to the virus, and Micieli et al. [57] demonstrated that *Cx. quinquefasciatus* from the USA showed an infection rate of 95.5% when fed with strain NY99-3356.

Regarding the titer analysis of body samples, our data showed that the variation in viral titer was directly related to the increase in dpi in G1, but the same did not occur in the G2 samples. The differences in the behavior of the virus titers in the two groups could have been influenced by a number of factors, including the strain of the mosquitoes, the difference in the proportion of blood plus virus provided to each of the groups, and the number of samples in each of the groups. Vogels et al. [35] demonstrated that higher temperatures increase the rate of WNV transmission by *Cx. pipiens,* and Richards et al. [58] also emphasizes the existence of complex relationships between environmental and biological factors that influence the susceptibility of mosquitoes to viruses, such as the age of the mosquito, the extrinsic incubation temperature, the dose of the virus, and the colony analyzed.

Comparison of oral infection of *Cx*. *quinquefasciatus* with two strains of WNV, WNV144 and NY99, at doses of 5.56 log10/5 µL and 3.88 log10/5 µL, respectively, found that lower oral doses reduced the proportion of mosquitoes that could be orally infected with the virus [59]. Additionally, in a study of field-collected *Cx*. *quinquefasciatus*, WNV was detected at a dose of 5.33 log10/5 µL [60]. In our study, the infectious dose was 7.84 log10/mL (7 × 10^7^ PFU/mL), mosquitoes behaved as expected, and virus was detected in body samples from dpi 7 because the infectious dose was higher than the minimum reported in other studies.

Our study obtained a dissemination rate of 13% at the 7th dpi, 33% at the 14th dpi, and 80% at the 21st dpi, indicating a trend of growth in the number of positive heads with increasing dpi, and the titer analysis showed there was a greater correlation between titer growth and increasing dpi. Richards et al. [58] emphasized the importance of considering environmental and biological interactions in analyses of vector competence, given the intra- and interpopulation variability in vector interactions with the environment.

We identified the presence of WNV in a saliva sample from the 7th dpi in the second experimental group but could not titrate the sample to a 1:10 dilution in the viral titration plate test. This observation may indicate a rapid dissemination of the virus in the vector organism, reaching the saliva region, but with a low viral titer, hampering its transmission by hematophagy. In the second experimental group (G2), a positive saliva sample was detected on the 14th dpi with a titer of 200 PFU/mL.

Saliva also showed a tendency to increase as dpi increased (4% at the 7th and 14th dpi, and 48% at the 21st dpi), but the correlation between the variables’ viral titer and day post infection was not statistically significant. Schneider et al. [61] emphasize that the action of intrinsic factors of the vectors influence the pathogenicity and virulence of infections, since salivary proteins can alter the trajectory of viral infection in mosquitoes, and Sanchez-Vargas et al. [62] highlights the action of the saliva gland infection and escape barriers (SGIB and SGEB, respectively) as modulators of arbovirus transmission.

In our study, viral titers in positive saliva were 200 PFU/mL on day 14 and on day 21 ranged from 100 PFU/mL to 3 × 10^6^ PFU/mL. These data corroborate findings from previous in vitro studies indicating that mosquitoes inoculated viral titers range from 10^1.2^ to 10^4.3^ PFU/mL [63,64]. However, a study using in vivo assays showed that *Cx*. *tarsalis* species inoculated an average of 10^4^–10^5^ PFU and *Cx*. *pipiens* 10^5.9^–10^6.1^ PFU, suggesting that viral doses inoculated into live hosts are higher than those obtained by artificial salivation [65].

## 5. Conclusions

This study showed that *Culex quinquefasciatus*, from Brazil, proved to be susceptible to artificial oral infection by the BEAN854747 strain of WNV and can be considered as a potential vector of WNV in Brazil.

It is notable that although arthropods have several tissue and immunological barriers that act to mitigate viral spread from the midgut to other tissues, the WNV strain analyzed was able to overcome such barriers, as we identified WNV in saliva samples on the 21st dpi in all groups analyzed.

We emphasize that the present study is the first conducted in Brazil to evaluate the susceptibility to oral infection and the vectorial competence of *Culex quinquefasciatus* mosquitoes for WNV transmission, considering that the virus has already been detected in several Brazilian states in different hosts, such as horses, domestic birds, humans, and, more recently, in a pool of *Culex* spp. collected in the Carajás region, southeastern Pará state, indicating the circulation of WNV in the country, thus demonstrating the risk of occurrence of arbovirus outbreaks, symptomatic cases of West Nile fever in humans, as well as the occurrence of zoonotic transmission cycles involving wild and domestic animals.

## Data Availability

The data presented in this study are available in the article.
